# Evaluation of Aligners and Root Resorption: An Overview of Systematic Reviews

**DOI:** 10.3390/jcm13071950

**Published:** 2024-03-27

**Authors:** Meiling Zhang, Peng Zhang, Jeong-Tae Koh, Min-Hee Oh, Jin-Hyoung Cho

**Affiliations:** 1 Department of Orthodontics, School of Dentistry, Chonnam National University, Gwangju 61186, Republic of Korea; 218451@jnu.ac.kr; 2 Department of Prosthodontics, School of Dentistry, Chonnam National University, Gwangju 61186, Republic of Korea; zhangpeng8883@jnu.ac.kr; 3 Department of Pharmacology and Dental Therapeutics, Hard-Tissue Biointerface Research Center, School of Dentistry, Dental Science Research Institute, Chonnam National University, Gwangju 61186, Republic of Korea; jtkoh@chonnam.ac.kr; 4 Department of Orthodontics, School of Dentistry, Dental 4D Research Institute, Dental Science Research Institute, Chonnam National University, Gwangju 61186, Republic of Korea; ohmh@jnu.ac.kr

**Keywords:** aligners, root resorption, AMSTAR 2, systematic review

## Abstract

**Background:** To evaluate the current evidence on clear aligners and root resorption using 3D and/or combined 2D and 3D methods from available systematic reviews and meta-analyses and to determine the relationship between root resorption and clear aligners using the AMSTAR 2 tool. **Methods:** A comprehensive literature search of systematic reviews investigating aligners and root resorption, published up until 31 December 2022, was conducted. The following electronic databases were searched: MEDLINE via PubMed, EMBASE, Google Scholar, Science Direct, Web of Science, Scopus, LIVIVO, and LILACS. There were no language restrictions. The inclusion criteria were restricted to studies focusing on root resorption utilizing either 3D methods exclusively or a combination of 2D and 3D techniques. Data were screened and analyzed for quality using the “A Measurement Tool to Assess Systematic Reviews (AMSTAR 2)” tool. Data extraction was conducted independently by two authors. The gathered information was categorized and synthesized narratively based on the primary findings elucidated within the reviews. **Results:** Out of a total of 1221 potentially eligible studies initially identified, 4 systematic reviews met the inclusion criteria following the exclusion of irrelevant studies. Among these, two systematic reviews (50%) were classified as low-quality, while the remaining two (50%) were deemed to be of critically low quality. **Conclusions:** Based on the findings of four systematic reviews, the root resorption rate was lower with the use of clear aligners than with fixed aligners. It is advisable to approach the interpretation of this conclusion with caution, as the quality of the available evidence is assessed to be very low. Higher quality systematic reviews are needed to substantiate this conclusion.

## 1. Introduction

Root resorption (RR) is a common and serious complication of orthodontics [1]. The frequency, prevalence, and gravity of root resorption escalate notably with the implementation of comprehensive fixed appliance orthodontic therapy [2,3]. Root resorption emerges as arguably one of the most lamentable complications within the domain of orthodontic practice. The incidence and severity of OIIRR are closely related to various factors, including the treatment duration, type of appliance, extraction and non-extraction, amount of orthodontic force, and type of tooth movement [4].

In recent years, compared with traditional fixed appliances, aligners have gradually gained popularity in orthodontics. Aligners have various advantages, including being invisible, removable, and more comfortable; promoting better periodontal health; facilitating easier oral hygiene maintenance; and they are less likely to disrupt eating [5,6]. Although the price is relatively expensive, more young people who pay attention to appearance are more willing to choose clear aligners as the method of orthodontic treatment. To date, the relationship between aligners and root resorption remains unclear. Some reports have suggested that the root resorption rate with aligners does not differ significantly from treatment with fixed appliances as the root apices are moved the same distances [7,8]. Conversely, other studies have reported that the root resorption rate of aligners is significantly lower than that of fixed appliances [9]. Still, other reports suggest that only the root resorption rate of the right maxillary central incisor is lowered in aligner treatment compared with in fixed appliance treatment, and that there are no differences in the other teeth [10]. Evidently, there is no consensus regarding the relationship between root resorption and aligners. Notably, systematic reviews are limited.

Importantly, the few systematic reviews available have concluded that current evidence is of low quality. For instance, the included studies used apical and panoramic radiographs for root resorption evaluations and measurements. Using a 2D method for analysis seriously underestimates the degree of root resorption. As root resorption occurs in all directions, evaluations using cone beam computed tomography (CBCT) offer a higher accuracy than two-dimensional methods [11,12]. Moreover, some systematic reviews did not adequately consider the study design [10]. Furthermore, some authors failed to consider the risk of bias for the articles included in their systematic reviews or did not use appropriate bias assessment tools [13,14]. These deficiencies have compromised the quality of existing systematic reviews. Recently, the “A Measurement Tool to Assess Systematic Reviews (AMSTAR 2)” tool has gained attention as a superior method for evaluating systematic reviews [15]. Hence, this article aimed to assess the available systematic reviews and meta-analyses focusing on the relationship between clear aligners and root resorption using either a 3D method and/or combined 2D and 3D methods, and to determine the relationship between root resorption and clear aligners using the AMSTAR 2 tool.

## 2. Materials and Methods

### 2.1. Study Protocol

Given the nature of the investigation, ethical approval was deemed unnecessary as it did not involve any intervention, patient participation, or the collection of personal data. This study rigorously adhered to the guidelines delineated in the Preferred Reporting Items for Systematic Review and Meta-Analyses (PRISMA) statement [16]. However, this review was not registered.

### 2.2. Eligibility Criteria

The eligibility criteria were established in accordance with the Population, Intervention, Comparison, Outcome, and Study (PICOS) framework, as delineated below [17].

Populations: Patients of any age undergoing orthodontic treatment for any type of malocclusion.

Intervention: Orthodontic treatment using any type of clear aligners.

Comparison: The orthodontic treatment involved the utilization of fixed orthodontic appliances of any type, or alternatively, an untreated control group was included for comparison.

Outcome: Evaluation of root resorption relative to clear aligner treatment (CAT) using 3D and/or combined 2D and 3D methods.

Study design: Systematic reviews with or without a meta-analysis. Studies employing any other design were excluded, and systematic reviews consisting solely of in vitro or animal studies were also omitted from consideration.

### 2.3. Information Sources, Search Strategy, and Study Selection

A comprehensive and extensive literature search was conducted, covering studies published up until 31 December 2022, using the following key terms: ‘root or tooth or apical resorption’, ‘aligners or Invisalign’, ‘orthodontics’, ‘systematic review’, and (or) ‘meta-analysis’. Eight electronic databases were searched (Table 1). No restrictions were applied concerning language, patient age range, publication status, and date. All pertinent articles were identified, retrieved, and independently evaluated for eligibility by M.L.Z. and M.H.O. Any disagreements were resolved through discussion with a third author (J.H.C.).

### 2.4. Data Items and Collection

Following the screening of eligible systematic reviews, the subsequent data extraction was carried out independently by the same two authors, and the process was repeated: 1. publication year, 2. study design, 3. number of included studies, 4. study type, 5. number of participants, 6. search period, 7. title of journal, 8. objective of study. Any disagreements were initially resolved through discussion with a third author (J.H.C.) as required to ensure consensus.

### 2.5. Quality Assessment

Both authors independently evaluated the included reviews based on the AMSTAR 2 quality assessment tool. Any discrepancies were initially addressed through discussion with the third author (J.H.C.), as required, to reach a consensus.

### 2.6. Data Synthesis

Data pooling was scheduled with the aim of quantitatively evaluating the impact of aligner treatment on root resorption, particularly in cases where clinical homogeneity was observed. To evaluate the homogeneity of the included reviews, various characteristics of the studies, including interventions, the methodologies applied for the detection and measurement of RR, and study design type, were considered. At the same time, in situations where clinical heterogeneity was identified, qualitative analysis was employed.

## 3. Results

### 3.1. Study Selection

A cumulative total of 1221 potentially eligible studies were identified during the screening phase. The initial screening of titles and abstracts reduced the number to 17 systematic reviews. After excluding the duplicates, six systematic reviews remained. Following the evaluation of the complete text, four were eligible for inclusion in this study and qualitatively analyzed.

### 3.2. Study Characteristics

**Figure 1:**
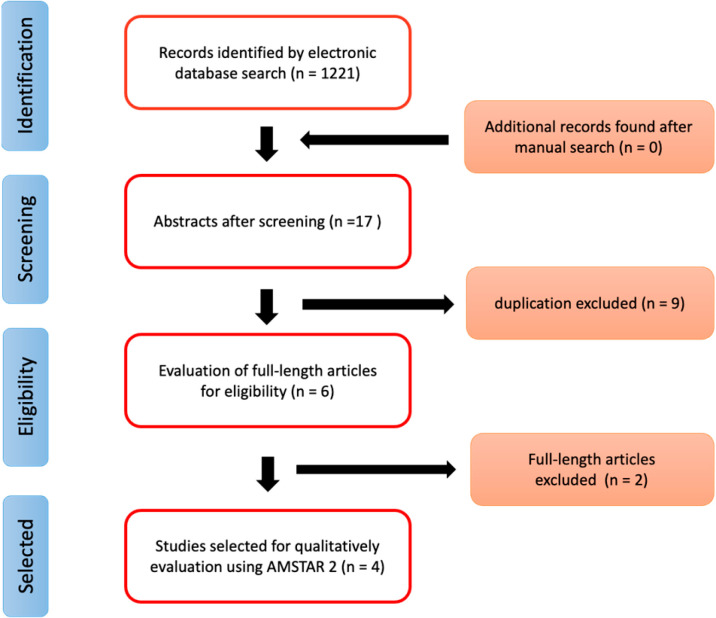
Flow diagram in this study.

Figure 1 presents the PRISMA flow diagram depicting the process of the literature selection. Additionally, a summary outlining the characteristics of the included systematic reviews can be found in Table 2. It is noteworthy that the encompassed studies were published up to the year 2022.

### 3.3. Quality of Evidence from the Data Synthesis

**Figure 2:**
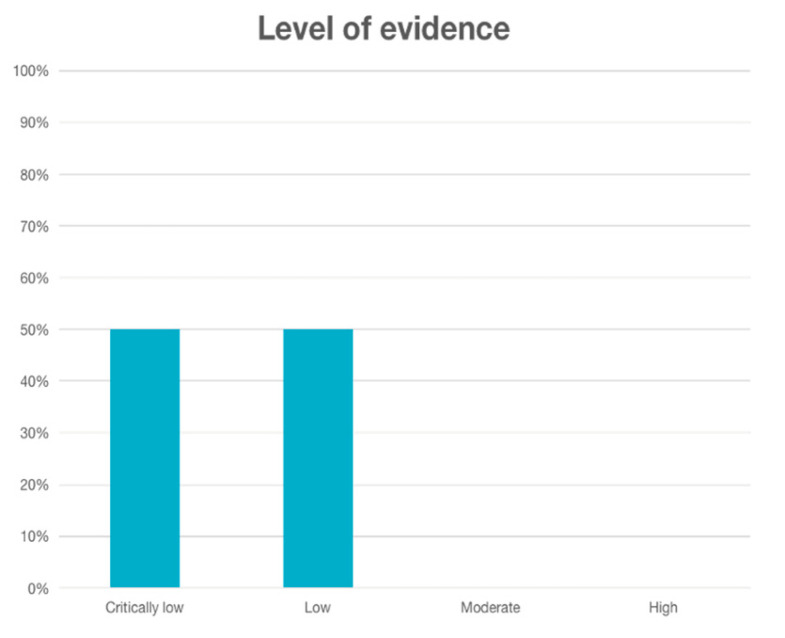
Level of evidence of systematic reviews included according to AMSTAR 2.

Based on the AMSTAR 2 checklist, the quality of the included reviews exhibited variability. Two studies (50%) were classified as low quality, while the other two reviews (50%) were considered of critically low quality (Table 3 and Table 4; Figure 2).

### 3.4. Data Synthesis

Subsequent meta-analysis was deemed unfeasible owing to the absence of primary data, standardized treatment protocols, and variations in interventions. Furthermore, clinical and methodological heterogeneity exist across the studies. Therefore, a qualitative synthesis was conducted by identifying the most important themes, and then, summarizing the findings accordingly.

### 3.5. Root Resorption According to the Detection Method

In total, 36 studies were included in the 4 systematic reviews. Of these, 7 evaluated root resorption by CBCT and 22 were evaluated by panoramic radiograph. Additionally, the original full text of the include article was not found in the 7 studies. From the systematic review by Vaibhav Gandhi and Shivam Mehta, CBCT showed a decreased magnitude of external apical root resorption (EARR) in 2D imaging, leading to the conclusion that 2D imaging might overstate the extent of EARR associated with orthodontic treatment [10].

### 3.6. Root Resorption in CAT versus Pre-Adjusted Edgewise Appliances (PEA)

All four articles compared the root resorption rate of the fixed appliance treatment versus aligner treatment for non-extraction cases. Two systematic reviews [10,18] included a meta-analysis, while the other two [13,14] were not quantitatively analyzed. From the available evidence, the occurrence and seriousness of RR were lower after CAT than with PEA [13]. The difference in the EARR of maxillary incisors in PEA versus CAT was not significant, except for the right maxillary central incisor (the PEA group showed significantly more EARR than the CAT group) [10]. Overall, the occurrence and severity of EARR were found to be lower in CAT compared with PEA [18]. The available evidence suggests that in cases of malocclusion not necessitating extractions, CAT was associated with a decreased likelihood of experiencing EARR compared with traditional fixed multi-bracket treatment [14].

## 4. Discussion

Root resorption is the process of the removal of cementum and/or dentine through physiological or pathological activity of tooth resorbing cells [19]. The cause of this pathological event is multifactorial, when the pressure generated by the appliance passing through the teeth exceeds the capillary pressure, the periodontal ligament will undergo ischemic necrosis, leading to the degeneration of the transparent zone, thereby activating tooth resorption cells and causing the loss of cementum and/or dentin [20,21]. OIIRR is likely to occur in most patients who have undergone orthodontic treatment [22]. As a high-risk factor for iatrogenic root resorption, orthodontic treatment deserves attention [23].

Advances in 3D printing orthodontic technology have substantially enhanced the treatment of non-extraction orthodontic cases. As a result, more clinicians and patients prefer CAT. In non-extraction cases, the incidence of EARR in CAT ranges between 41.81–68.3% (including 2.83–6.31% severe resorption). Almost all patients have at least 1–2 teeth affected by root resorption [24]. Of the 4 systematic reviews included in our study, 3 were non-extraction studies. Additionally, of the 11 studies included in the remaining systematic review, 6 were non-extraction cases and 7 were tooth extraction cases. For non-extraction cases, aligners have obvious advantages. However, in cases with severe crowding, certain technical difficulties limit the usefulness of CAT. In the four included systemic reviews, EARR was most frequently detected in the upper and lower anterior teeth. Notably, only a few studies included canines, premolars, and first molars. At present, the available research on root resorption in CAT is mostly limited to non-extraction cases. Most studies have focused on anterior teeth. Studies including posterior teeth are limited [25].

Aligners are removable appliances and must be removed when eating and brushing teeth. Some patients have shown poor compliance during treatment, failing to wear the aligners for the necessary duration. Consequently, tooth movement efficiency is compromised, deviating from the original design plan and resulting in the need to redesign the treatment plan. With CAT, the interval is longer, so the orthodontic force generated tends to be discontinuous, and the time of force application is shortened, which, in turn, reduces the risk of EARR. Consequently, the cementum is repaired to varying degrees during the non-stressed periods [26]. With CAT, each tooth movement step is precisely designed. Compared with fixed orthodontic appliances, reciprocating tooth movements are avoided to a great extent. Tooth movement reciprocation is not only a high risk for root resorption [27], but can also prolong the treatment time, thereby increasing the risk of root resorption [28].

Individualized treatment planning is essential in clear aligner therapy to achieve desired orthodontic outcomes while minimizing the risk of root resorption [29]. Extensive malocclusions, including crowding, spacing, and misalignment, may require more extensive tooth movements and longer treatment durations. Orthodontists need to carefully evaluate the pretreatment extent of malalignment to develop personalized treatment plans that address specific orthodontic concerns effectively.

Barbagallo et al. compared the root resorption of the premolars under light force (25 g) and gravity (225 g) for 8 weeks, comparing ClearSmile^®^ aligners and traditional fixed orthodontics. The results of that study demonstrated that root resorption in the clear aligner group was basically similar to that of the fixed orthodontic light force group, suggesting that clear aligners are associated with lighter forces [30], and light forces reduce the risk of EARR [31]. For non-extraction cases with mild to moderate crowding, the anterior teeth often undergo interproximal reduction (IPR) as part of CAT, which reduces the moving distance of the anterior teeth and shortens the treatment duration. This may contribute to lowering the risk and severity of root resorption. Unlike fixed orthodontics, where force is applied at the center of the crown, aligners apply force through the appliance itself, as well as the accessories. This may be one of the reasons for the lower risk of root resorption in CAT. Further investigations are required to verify this hypothesis.

AMSTAR, developed in 2007, is currently the most popular extensive tool for evaluating systematic reviews that include randomized controlled trials [32]. However, with the increasing number of non-randomized controlled trials, AMSTAR has shown certain limitations. Consequently, a working group has been established to further develop the tool. In 2017, AMSTAR 2, which is indicated also for non-randomized controlled studies, was launched. This new version aligns with the PICO framework better. It also facilitates sufficiently detailed assessments of biases in included studies, including bias in statistical pooling and bias in result interpretation and discussion [32].

There are many systematic reviews on root resorption, but they are largely based on 2D studies. In this study, the systematic reviews included 3D and 2D studies were selected (because systematic reviews that included 3D methods for measurement were not all included). The selection involved several layers of screening. In the end, only four systematic reviews were eligible for inclusion. These were evaluated using the AMSTAR 2 method. The outcome indicated that none of the included systematic reviews were of high quality. For studies that investigated root resorption with CAT, there were limited studies that analyzed EARR using CBCT and compared the results with those detected by panoramic and apical radiographs. Notably, apical radiographs underestimated root lengths by an average of 2.6 mm, whereas CBCT underestimated root lengths by less than 0.3 mm [33]. The use of CBCT to detect root resorption is increasing and should be promoted. To draw accurate conclusions, high-quality randomized controlled studies for inclusion in systematic reviews and meta-analyses are paramount. Summarizing the findings from the four included systemic reviews, current evidence suggests that while aligners cannot prevent root resorption in orthodontic treatment, the incidence of root resorption is reduced relative to fixed orthodontic treatment. Our result is roughly consistent with the conclusions of previous studies [34]. Importantly, Vaibhav Gandhi et al., in their meta-analysis, concluded that the right maxillary central incisor is not affected by the type of treatment appliances and that there is no difference in the root resorption rate. This conclusion was not consistent with the other three systematic reviews.

## 5. Limitations

The number of systematic reviews that met the inclusion criteria was small. The evidence suggests that all four were of low or critically low quality. Without high-quality studies, a meta-analysis could not be performed as the risk of bias would be high.

Currently, there are almost no systematic reviews focusing solely on CBCT studies, and the number of 3D studies included in existing systematic reviews is relatively small.

Most of the studies are non-extraction cases, and very few tooth extraction cases have been investigated. For cases with severe crowding and orthodontically complicated cases, the technical support for CAT needs to be further improved.

## 6. Conclusions

In mildly to moderately crowded non-extracted cases, root resorption was lower with clear aligners therapy compared with fixed orthodontic treatment. This conclusion warrants cautious interpretation due to the very low quality of the available evidence. In the future, higher quality systematic reviews are needed to substantiate this thesis.

Evaluating root resorption with 2D methods may result in underestimations as root resorption occurs in all directions. To accurately assess root resorption, CBCT is recommended.

## Figures and Tables

**Table 1:** Root resorption and clear aligner treatment.

Database	Search Strategy	Sum
1 MEDLINE (via Pubmed)	(root or tooth or apical) * resorption and (aligners or invisalign) and orthodontics	159
2 Google Scholar	(root or tooth or apical) * resorption and (aligners or invisalign) and orthodontics	678
3 ScienceDirect	((root or tooth or apical) * resorption) and (aligners or invisalign)	55
4 Web of Science	(root resorption) and orthodontics	39
5 Embase	(‘root resorption’/exp OR ‘root resorption’ OR ((‘root’/exp OR root) AND (‘resorption’/exp OR resorption))) AND (aligners OR ‘invisalign’/exp OR invisalign)	251
6 Scopus	(root or tooth or apical) * resorption and (aligners or invisalign) and orthodontics	error
7 LIVIVO	root resorption and aligner and review	36
8 LILACS	(root or tooth or apical) * resorption and (aligners or invisalign) and orthodontics	3
Total		1221

**Table 2:** List of systematic reviews.

Author	Year	Study Design	No. of Study	No. of Participants	Type of Study	Period of Search	Journal	Objective	Quality of Evidence
Vaibhav Gandhi et al. [10]	2021	systematic review and meta-analysis	16	523	4 prospective, 12 retrospective	up to 31 December 2019	Eur J Orthod	tTo evaluate and compare the amount of EARR observed during the orthodontic treatment with PEA or CAT and with 2D or 3D methods	Low
Sadauskaitė U, Berlin V. [13]	2020	systematic review	6	686	2 retrospective, 1 prospective, 1 pilot, 1 case-control, 1 NRCT	2009 to 2019	Med. Sci	To evaluate the link between clear aligner therapy and EARR and to the amount of EARR using clear aligner therapy and fixed orthodontic treatment	Critically low
Xuanwei Fang et al. [18]	2019	systematic review and meta-analysis	11	828	6 before-and-after, 4 cohort	up to December 2018	Orthod Craniofac Res	To investigate the EARR in participants receiving CAT and it with PEA	Low
Rajae Elhaddaoui et al. [14]	2016	systematic review	3	217	1 NRCT, 1 retrospective, 1 RCT	up to December 2015	Int orthod	To assess the incidence and severity of RR following CAT and associated factors, a comparative analysis also made with fixed multi-bracket treatments	Critically low

EARR: external apical root resorption. CAT: clear aligner treatment. PEA: pre-adjusted edgewise appliance. RR: root resorption. RCT: randomized controlled trial. NRCT: non-randomized controlled trial.

**Table 3:** Items of the “A Measurement Tool to Assess Systematic Reviews” (AMSTAR 2) tool [15].

	Meeting the Criteria		
	Yes	Partial Yes	No
1. Did the research questions and inclusion criteria for the review include the components of PICO?	4		
2. Did the report of the review contain an explicit statement that the review methods were established prior to the conduct of the review and did the report justify any significant deviations from the protocol?	2		2
3. Did the review authors explain their selection of the study designs for inclusion in the review?			4
4. Did the review authors use a comprehensive literature search strategy?	1	2	1
5. Did the review authors perform study selection in duplicate?	2		2
6. Did the review authors perform data extraction in duplicate?	2		2
7. Did the review authors provide a list of excluded studies and justify the exclusions?	1		3
8. Did the review authors describe the included studies in adequate detail?		4	
9. Did the review authors use a satisfactory technique for assessing the risk of bias in individual studies that were included in the review?	2		2
10. Did the review authors report on the sources of funding for the studies included in the review?	1		3
11. If meta-analysis was performed did the review authors use appropriate methods for statistical combination of results?	1		1
12. If meta-analysis was performed, did the review authors assess the potential impact of risk of bias in individual studies on the results of the meta-analysis or other evidence synthesis?	2		2
13. Did the review authors account for risk of bias in individual studies when interpreting/discussing the results of the review?	1		3
14. Did the review authors provide a satisfactory explanation for, and discussion of, any heterogeneity observed in the results of the review?	2		
15. If they performed quantitative synthesis did the review authors carry out an adequate investigation of publication bias (small study bias) and discuss its likely impact on the results of the review?	3		1
16. Did the review authors report any potential sources of conflict of interest, including any funding they received for conducting the review?	2		2

AMSTAR 2 categorizes the level of evidence according to the following: High: No or one non-critical weakness: the systematic review provides an accurate and comprehensive summary of the results of the available studies that address the question of interest. Moderate: More than one non-critical weakness *: the systematic review has more than one weakness but no critical flaws. It may provide an accurate summary of the results of the available studies that were included in the review. Low: One critical flaw with or without non-critical weaknesses: the review has a critical flaw and may not provide an accurate and comprehensive summary of the available studies that address the question of interest. Critically low: More than one critical flaw with or without non-critical weaknesses: the review has more than one critical flaw and should not be relied on to provide an accurate and comprehensive summary of the available studies. * Multiple non-critical weaknesses may diminish confidence in the review and it may be appropriate to move the overall appraisal down from moderate to low confidence.

**Table 4:** The quality of evidence according to AMSTAR 2 [15].

	[10]	[13]	[18]	[14]
1. Did the research questions and inclusion criteria for the review include the components of PICO?	Y	Y	Y	Y
2. Did the report of the review contain an explicit statement that the review methods were established prior to the conduct of the review and did the report justify any significant deviations from the protocol?	Y	N	Y	N
3. Did the review authors explain their selection of the study designs for inclusion in the review?	N	N	N	N
4. Did the review authors use a comprehensive literature search strategy?	Y	N	PY	PY
5. Did the review authors perform study selection in duplicate?	Y	N	Y	N
6. Did the review authors perform data extraction in duplicate?	Y	N	Y	N
7. Did the review authors provide a list of excluded studies and justify the exclusions?	N	N	Y	N
8. Did the review authors describe the included studies in adequate detail?	PY	PY	PY	PY
9. Did the review authors use a satisfactory technique for assessing the risk of bias in individual studies that were included in the review?	Y	N	Y	N
10. Did the review authors report on the sources of funding for the studies included in the review?	N	Y	N	N
11. If meta-analysis was performed did the review authors use appropriate methods for statistical combination of results?	Y	NM	N	NM
12. If meta-analysis was performed, did the review authors assess the potential impact of risk of bias in individual studies on the results of the meta-analysis or other evidence synthesis?	N	NM	N	NM
13. Did the review authors account for risk of bias in individual studies when interpreting/discussing the results of the review?	Y	N	Y	N
14. Did the review authors provide a satisfactory explanation for, and discussion of, any heterogeneity observed in the results of the review?	Y	N	Y	N
15. If they performed quantitative synthesis did the review authors carry out an adequate investigation of publication bias (small study bias) and discuss its likely impact on the results of the review?	Y	NM	N	N
16. Did the review authors report any potential sources of conflict of interest, including any funding they received for conducting the review?	Y	Y	Y	Y
Quality of evidence	Low Critically	Low Critically	low	low

Y: yes; N: no; PY: partial yes; NM: no meta-analysis conducted.

## Data Availability

Not applicable.
